# Identification of a novel HOOK3-FGFR1 fusion gene involved in activation of the NF-kappaB pathway

**DOI:** 10.1186/s12935-022-02451-y

**Published:** 2022-01-26

**Authors:** Xuehong Zhang, Furong Wang, Fanzhi Yan, Dan Huang, Haina Wang, Beibei Gao, Yuan Gao, Zhijie Hou, Jiacheng Lou, Weiling Li, Jinsong Yan

**Affiliations:** 1 Department of Hematology, Liaoning Medical Center for Hematopoietic Stem-Cell Transplantation, Liaoning Key Laboratory of Hematopoietic Stem-Cell Transplantation and Translational Medicine, Dalian Key Laboratory of Hematology, the Second Hospital of Dalian Medical University, 116027 Dalian, China; 2 Diamond Bay Institute of Hematology, the Second Hospital of Dalian Medical University, 116027 Dalian, China; 3 Institute of Cancer Stem Cell, Dalian Medical University, 116044 Dalian, China; 4 Department of Neurosurgery, The Second Affiliated Hospital of Dalian Medical University, 116044 Dalian, China; 5 Department of Biotechnology College of Basic Medical Science, Dalian Medical University, 116044 Dalian, China

**Keywords:** 8p11 myeloproliferative syndrome, HOOK3-FGFR1, RNA sequencing, NF-kappaB signaling pathway, Expression signature

## Abstract

**Background:**

Rearrangements involving the fibroblast growth factor receptor 1 (FGFR1) gene result in 8p11 myeloproliferative syndrome (EMS), which is a rare and aggressive hematological malignancy that is often initially diagnosed as myelodysplastic syndrome (MDS). Clinical outcomes are typically poor due to relative resistance to tyrosine kinase inhibitors (TKIs) and rapid transformation to acute leukemia. Deciphering the transcriptomic signature of FGFR1 fusions may open new treatment strategies for FGFR1 rearrangement patients.

**Methods:**

DNA sequencing (DNA-seq) was performed for 20 MDS patients and whole exome sequencing (WES) was performed for one HOOK3-FGFR1 fusion positive patient. RNA sequencing (RNA-seq) was performed for 20 MDS patients and 8 healthy donors. Fusion genes were detected using the STAR-Fusion tool. Fluorescence *in situ* hybridization (FISH), quantitative real-time PCR (qRT-PCR), and Sanger sequencing were used to confirm the HOOK3-FGFR1 fusion gene. The phosphorylation antibody array was performed to validate the activation of nuclear factor-kappaB (NF-kappaB) signaling.

**Results:**

We identified frequently recurrent mutations of ASXL1 and U2AF1 in the MDS cohort, which is consistent with previous reports. We also identified a novel in-frame HOOK3-FGFR1 fusion gene in one MDS case with abnormal monoclonal B-cell lymphocytosis and ring chromosome 8. FISH analysis detected the FGFR1 break-apart signal in myeloid blasts only. qRT-PCR and Sanger sequencing confirmed the HOOK3-FGFR1 fusion transcript with breakpoints located at the 11th exon of HOOK3 and 10th exon of FGFR1, and Western blot detected the chimeric HOOK3-FGFR1 fusion protein that is presumed to retain the entire tyrosine kinase domain of FGFR1. The transcriptional feature of HOOK3-FGFR1 fusion was characterized by the significant enrichment of the NF-kappaB pathway by comparing the expression profiling of FGFR1 fusion positive MDS with 8 healthy donors and FGFR1 fusion negative MDS patients. Further validation by phosphorylation antibody array also showed NF-kappaB activation, as evidenced by increased phosphorylation of p65 (Ser 536) and of IKBalpha (Ser 32).

**Conclusions:**

The HOOK3-FGFR1 fusion gene may contribute to the pathogenesis of MDS and activate the NF-kappaB pathway. These findings highlight a potential novel approach for combination therapy for FGFR1 rearrangement patients.

**Supplementary Information:**

The online version contains supplementary material available at 10.1186/s12935-022-02451-y.

## Background

8p11 myeloproliferative syndrome (EMS), which is characterized by translocation of the fibroblast growth factor receptor-1 (FGFR1) gene at the 8p11-12 chromosome locus, is recognized as a distinct entity in 2016 World Health Organization (WHO) classification [1, 2]. EMS patients may be initially diagnosed as myelodysplastic syndromes (MDS) and typically present with bilineage disease (myeloid and lymphoid) and rapid progression to acute myeloid leukemia (AML, ~80%) or T- or B-cell lymphomas [1]. These patients are resistant to current therapeutic regimens including tyrosine kinase inhibitors (TKIs) and have a 5-year survival rate of < 20% [2, 3]. Currently, allogeneic hematopoietic stem-cell transplantation is the only potentially curative therapeutic option to prolong survival [3, 4]. Thus, there is an urgent need for alternative treatment plans for patients who are either awaiting or unable to receive transplantation.

To date, at least 16 different partner genes of FGFR1 fusion have been identified: ZMYM2 [5–7], FGFR1OP [8], CNTRL [9], ERVK3-1 [10], BCR [11, 12], NUP98 [13], FGFR1OP2 [14], TRIM24 [15], MYO18A [16], CPSF6 [17], LRRFIP1 [18], CUX1 [19], TPR [20], RANBP2 [21], SQSTM1 [22], and TFG [23]. Among them, ZMYM2 and BCR are the most common partner genes [1]. Although all FGFR1 rearrangement cases consistently show constitutive activation of FGFR1 kinase, substantial heterogeneity of clinical presentation is exhibited depending on the specific nature of the partner gene [1, 23, 24]. HOOK3 is an adaptor protein with roles in microtubule-dependent intracellular vesicles and protein trafficking. A high level of HOOK3 expression is associated with poor prognosis in prostate cancer [25]. However, HOOK3 has not been reported to form oncogenic fusions as the 5’ partner with FGFR1 in hematological malignancies.

Previous research has demonstrated that FGFR1 fusion protein can activate NOTCH1 signaling or tyrosine phosphorylation of downstream targets, such as FLT3, MYC, and STAT5, in human cells and mouse models [26–29]. Highly expressed FGFR1 has the potential to promote nuclear factor-kappaB (NF-kappaB) signaling in cancer [30, 31]. However, the relationship between the FGFR1 fusion gene and NF-kappaB pathway remains unclear. In the present study, we performed RNA sequencing (RNA-seq) of 20 MDS patients and identified a novel in-frame HOOK3-FGFR1 fusion gene in one MDS case accompanied by abnormal monoclonal B-cell lymphocytosis. We then validated this finding with the structure of HOOK3 exons 1-11 joining to FGFR1 exons 10-18, and Western blot confirmed the presence of chimeric HOOK3-FGFR1 fusion protein. We also observed significant enrichment of NF-kappaB signaling as the transcriptomic signature in the FGFR1 fusion positive case compared with the healthy donors and FGFR1 fusion negative cases. Furthermore, phosphorylated p65, IKB-alpha, and TAK1 were shown to be up-regulated in HOOK3-FGFR1 cells based on the RayBiotech NF-kappaB pathway phosphorylation arrays. The NF-kappaB activation induced by HOOK3-FGFR1 fusion provides potential target for combination therapy of FGFR1 rearrangement patients.

## Materials and methods

### Patients and samples

We collected bone marrow (BM) samples from MDS patients (n = 20) and healthy donors (n = 8) between February 1, 2019 and June 10, 2021. Detailed clinical information about the MDS patients is summarized in Additional file 1: Table S1. This study was approved by the Research Ethics Board of the Second Hospital of Dalian Medical University and was performed in accordance with the Declaration of Helsinki. Written informed consent was obtained from all participants.

### Cytogenetics and fluorescence in situ hybridization (FISH)

The FISH technique was used for karyotype analysis following standard clinical protocols. Briefly, 200 interphase cells and 200 metaphase cells were analyzed for disruptions in FGFR1. The nuclei were probed using the FGFR1 Break-apart/Amplification probe (LPS018, CytoCell, UK), which comprises a green 272 kb probe and a red 267 kb probe positioned on the 3’ and 5’ ends of the FGFR1 gene, respectively. We considered a case to be positive when > 15% of cells displayed separation signals.

### Immunophenotyping

For the bone marrow samples of HOOK3-FGFR1 positive patient, we analyzed the bone marrow using flow cytometry with a mixed set of monoclonal antibodies. The panel of antibodies included: CD45-PerCP, CD117-APC, anti-HLA-DR-APC, CD34-PE, CD38-FITC, CD11b-APC, CD13-PE, CD33-PE, CD14-FITC, CD15-FITC, CD16-FITC, CD41a-FITC, CD42b-PE, CD61-FITC, CD36-FITC, CD64-PE, CD71-APC, CD300e-APC, CD235a-PE, CD10-PE, CD19-PerCP, CD20-PE, CD22-FITC, cCD79a-PE, cAnti-Lambda-PE, cAnti-Kappa-FITC, CD138-APC, CD56-PacificBlue-A, CD3-APC or CD3-FITC, cytoplasmic CD3 (cCD3-APC), CD4-PE, CD5-FITC, CD7-FITC. The data were acquired and analyzed using a FACS BD LSR Fortessa flow cytometer with the aid of FACSDiva software (v8.0.1, Becton Dickinson, San José, CA, USA).

### RNA/DNA extraction, library preparation, and sequencing

DNA and total RNA were extracted from cryopreserved mononucleated cells (MNCs) using the All Prep DNA/RNA Mini Kit (Qiagen Company, Cat. 80,204). RNA concentration and purity were measured using a Qubit 2.0 Fluorometer (Life Technologies) and Bioanalyzer 2100 (Agilent Technologies).

Libraries were prepared according to the protocol of the TruSeq RNA/TruSeq DNA Sample Preparation Kit (Illumina) and the library quality was assessed using Bioanalyzer 2100 (Agilent Technologies). Massively parallel RNA-seq and whole exome sequencing (WES) were performed on a NovaSeq platform with paired-end 150 bp read-length by the Novogene Company (Beijing, China).

### Mutation analysis

Target mutation analysis was performed on the patient samples (n = 20) using a panel of 38 commonly mutated genes in myeloid hematologic malignancies. The full list of the tested genes is available in Additional file 1: Table S2. For the HOOK3-FGFR1 positive patient, we identified variants from the WES data under the GATK pipeline and annotated the variants using ANNOVAR. All mutations detected by WES and target sequencing are listed in Additional file 1: Table S3.

### Detection of fusion transcripts and RNA expression analysis

We applied STAR-Fusion to detect the fusion transcripts (Additional file 1: Table S4). For gene expression, we mapped the sequencing data to the reference genome (hg38) using STAR [32] and defined the transcript coordinates according to the gene annotation format file (GTF file) from GENCODE (Release 27, GRCh38). The gene expression abundances are reported as Reads Per Kilobase per Million mapped reads (RPKM) obtained using the Cufflinks package [33]. The DESeq2 package from R (http://cran.r-project.org/) was used to obtain the differential expressed genes between the HOOK3-FGFR1 positive samples and normal samples (Additional file 1: Table S5).

### Pathway enrichment analysis

We selected the gene set enrichment analysis (GSEA) using the JAVA program (http://software.broadinstitute.org/gsea/index.jsp) as the enrichment tool for data analysis [34]. The molecular pathways correlated to HOOK3-FGFR1 fusion were identified by conducting 5,000 permutations using the Molecular Signatures Database (MSigDB). Statistical over-representation and enrichment gene sets were considered with nominal p-values ≤ 0.01.

### Quantitative real-time PCR (qRT-PCR) and Sanger sequencing

PCR amplification was performed using the following primers: HOOK3 forward: 5′-GATCGACGTGCTGAGACA-3′ and FGFR1 reverse: 5′-CAACACCACCTGCCCAAA-3′. The PCR products were analyzed by 1% agarose gel electrophoresis at 110 V for 35 min and purified using a DNA Purification Kit (EasyPure® Quick Gel Extract Kit). Sanger sequencing was performed using the same primers (Sangon Biotech, Shanghai, China).

### Cell culture and transfections

HEK293T cells were cultured in RPMI 1640 medium (Gibco, USA) with 10% fetal bovine serum (FBS, ExCell Bio, China). The cells were then incubated at 37 ℃ in 5% CO_2_. We amplified the full-length coding sequence of the HOOK3-FGFR1 fusion transcript from the primary patient (Case 1) and cloned it into the LVX-IRES-puro Vector (ShanghaiHarmonious One Biotechnology Co., Ltd, China). We used the liposome transfection method to transfect the lentiviral constructs with packaging plasmids PSPAX2 and PMD2G into HEK293 T cells to produce replication-deficient viruses. The supernatant was harvested 72 h later and riboprotein was produced by transiently transfecting 293T cells. After 72 h, the transfection efficiency was evaluated by Western blot using the FLAG antibody (#66008-3-Ig, Proteintech Group).

### Phosphorylation antibody array

293 T Vector/HOOK3-FGFR1 (5 × 10^5^) cells were plated in 10 cm dishes. Cells were then collected and the lysate was extracted. After dilution at 500 µg/ml with blocking buffer, the lysates were analyzed using a commercial NF-kappaB pathway phosphorylation antibody array (Cat: #AAH-NFKB-1-2; RayBiotech, Norcross, GA, USA) according to the manufacturer’s instructions. Briefly, the membranes were blocked with blocking buffer for 30 min at room temperature and incubated with 2 mL of the supernatants (diluted 1:2 in blocking buffer) for 2 h at room temperature. After washing, a biotin-conjugated antibody detection cocktail was added and incubated overnight at 4 °C, followed by an additional overnight incubation at 4 °C with streptavidin-conjugated peroxidase at room temperature. The membranes were then incubated with peroxidase substrate and the results were documented using XAR films. The chemiluminescence signaling intensity was quantified using Quantity One software (Bio-Rad).

## Results

### The mutation landscape of 20 MDS patients

**Fig. 1:**
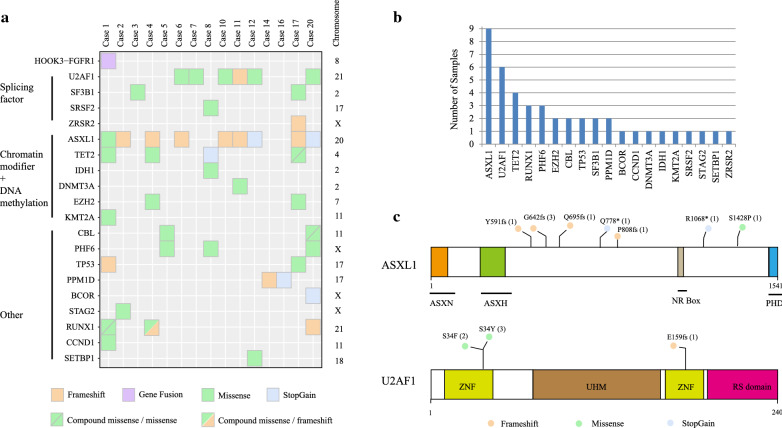
Specific sequencing data of 20 patients with MDS. **a** Summary of mutation data in the MDS cohort. **b** The bar plot shows the number of specific mutations in our MDS cohort. **c** Functional structures and mutations of ASXL1 and U2AF1 proteins. ASX: Asx homology, ASXN: Asx N-terminal, NR box: nuclear receptor co-regulator binding motif, PHD: plant homeodomain, ZNF: zinc finger, UHM: U2AF homology motif.

As EMS is often initially diagnosed as MDS, we performed DNA and RNA sequencing for a cohort of 20 MDS patients. The median age of these patients is 59 years (range, 13-78 years), and the male:female ratio is 1:1. Clinical data for all patients are available in Additional file 1: Table S1. For Case 1 who was diagnosed as MDS with abnormal monoclonal B-cell lymphocytosis, RNA-seq detected a novel in-frame HOOK3-FGFR1 fusion gene (Additional files 1: Table S4). Thus, we also performed WES to detect additional mutations in Case 1, upon the target DNA panel including 38 commonly mutated genes in myeloid hematologic malignancies (Additional files 1: Table S2). The mutations identified in all patients are presented in Fig. 1a with further details provided in Additional file 1: Table S3. In our MDS cohort, 19 different genes were mutated: ASXL1 (9/20, 45%); U2AF1 (6/20, 30%); TET2 (4/20, 20%); RUNX1 (3/20, 15%); PHF6 (3/20, 15%); SF3B1 (2/20, 10%), CBL (2/20, 10%), TP53 (2/20, 10%), EZH2 (2/20, 10%), PPM1D (2/20, 10%) and CCND1, DNMT3A, IDH1, KMT2A, SETBP1, STAG2, BCOR, ZRSR2 and SRSF2 (1/20, 5% each) (Fig. 1b). In our cohort, we identified nine mutations in the ASXL1 gene (six frameshift, one missense, and two stopgain mutations), although no mutations were located in the functional domain of ASXL1 (Fig. 1c). In contrast, all six variants of U2AF1 were located in the key zinc finger domain (Fig. 1c). The recurrently mutated genes involve in ASXL1 and U2AF1 in our MDS cohort which is consistent with previous study [35].

### Clinical presentation of one EMS patient with ring chromosome 8

**Fig. 2:**
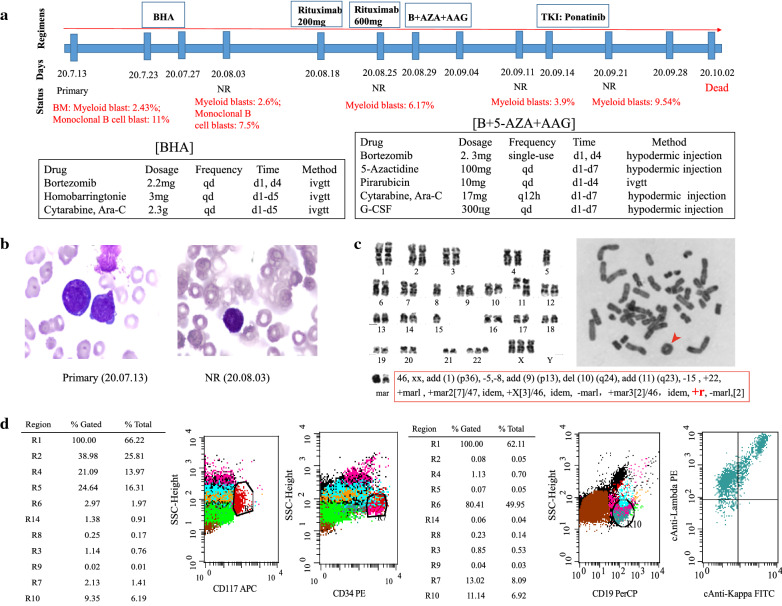
Clinical presentation of the patient with the HOOK3-FGFR1 fusion gene. **a** The entire treatment process of the HOOK3-FGFR1 positive patient. **b** Wright stain of bone marrow aspirate smear from Case 1, with blast cells clearly seen in the primary and NR specimens. **c** Karyotype analysis showed complex abnormalities and one additional ring chromosome (indicated by the red arrow). **d** Flow cytometry analysis of immunophenotypic markers for the HOOK3-FGFR1 positive patient. Total cells are gated on SSC/FSC plot where viable cells are selected for following analysis. The bone marrow cells of this patient were labeled with monoclonal antibody CD45. The diagnosis stage flow results showed the blast cells were positive for CD117 and CD34 (myeloblasts: 2.43%), and positive for CD19 and cLambda (monoclonal B-lymphoid cells: 11%)

The clinical course of Case 1 was shown in Fig. 2a. This patient is a 58-year-old woman who suddenly presented with unconsciousness after initial complaints of fatigue and chest tightness dyspepsia, abdominal distention, and early satiety lasting for 1 month. Blood tests revealed a leukocytosis (white blood cell: 4 × 10^9^/L), anemia (hemoglobin: 29 g/L), and thrombocytopenia (platelet: 4 × 10^9^/L). The BM wright-stained smear showed decreased myelodysplasia, with 3.5% myeloblasts (Fig. 2b). Cytogenetic analysis revealed the following complex karyotype: 46,xx,add(1)(p36),-5,-8,add(9)(p13),del(10)(q24),add(11)(q23),-15,+22,+marl,+mar2 [7]/47,idem,+X [3]/46,idem,-marl,+mar3 [2]/46,idem,+r,-marl [2] (Fig. 2c). We also observed an abnormal ring chromosome (RC), which indicates poor prognosis (Fig. 2c). Immunophenotyping by flow cytometry (FCM) identified 2.43% myeloblasts (positive for CD34 and CD117) and 11% monoclonal B-lymphoid cells (positive for CD19 and cLambda) (Fig. 2d). Multiplex RT-PCR analysis for 43 leukemia-related fusion genes showed a negative result (data not shown). Target DNA sequencing of 38 genes (Additional file 1: Table S2) and WES revealed mutations in the TET2, ASXL1, KMT2A, RUNX1, TP53, and CCND1 genes (Fig. 1a, Additional file 1: Table S4). RNA-seq detected a novel HOOK3-FGFR1 fusion gene involved in 8p11 locus, thus this patient was diagnosed as EMS based on the WHO 2016 criteria.

Initially, the patient received standard induction chemotherapy of the BHA regimen (bortezomib, homobarringtonie, and cytarabine), but showed no remission (NR) with 2.6% myeloid blasts and 7.5% monoclonal B-lymphoid blasts (Fig. 2a). The patient was sequentially treated with rituximab 200 mg and rituximab 600 mg within one week. The FCM results indicated the disappearance of monoclonal B-lymphoid blasts, but 6.17% myeloid blasts remained (Fig. 2a). As further treatment, one course of B + 5-AZA+AAG regimen (bortezomib, 5-azacytidine, pirarubicin, cytarabine, and granulocyte colony stimulating factor (G-CSF)) was given, and 3.9% myeloid blasts indicated NR (Fig. 2a). As ponatinib has been proven to be effective in the treatment of FGFR1 fusion positive patients [36–38], ponatinib treatment was started. However, this patient failed to respond to 1 week of ponatinib treatment (Fig. 2a). The patient died of pulmonary infection on October 2, 2020.

### The confirmation and feature of a novel HOOK3-FGFR1 fusion gene

**Fig. 3:**
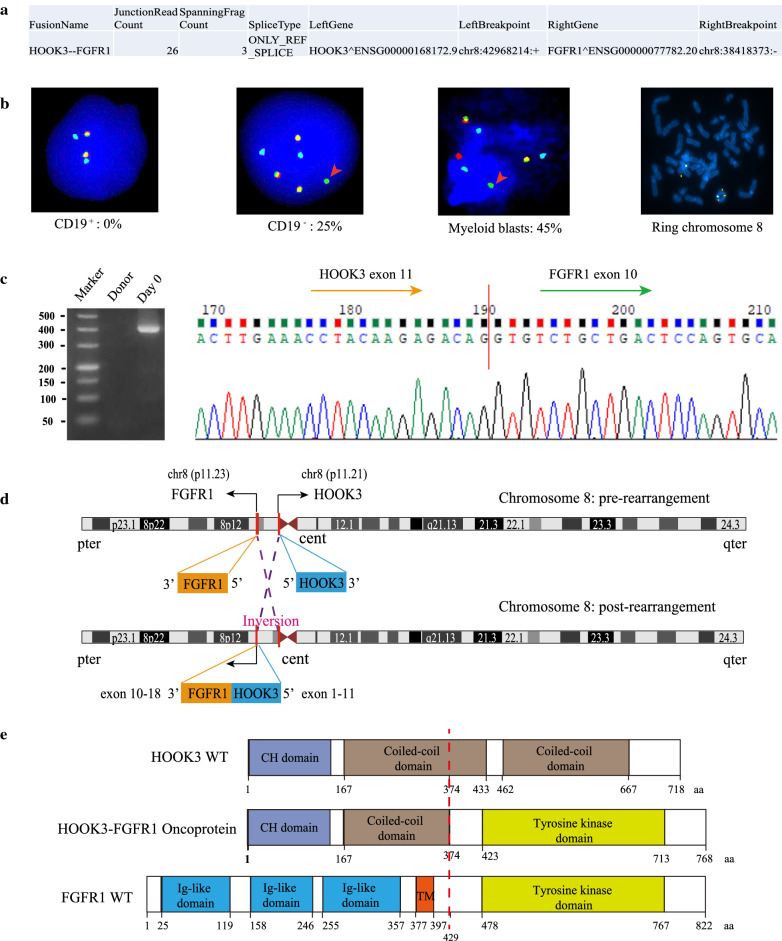
Validation and characterization of a novel HOOK3-FGFR1 fusion. **a** RNA sequencing analysis result revealed the chromosome positions of breakpoints in HOOK3 and FGFR1. **b** Interphase FISH analysis with FGFR1/D8Z2 Breakapart/Amplification probe (LPS018, CytoCell, UK) revealed a split FGFR1 signal pattern in the CD19- population and Myeloid blasts. The 5′ and 3′ FGFR1 are labeled with red and green, respectively; the D8Z2 (8p11-q11) region is labeled with blue as the control signal. The red arrow indicates a break-apart signal in the FGFR1 gene, and the percentages of positive signal detected in the bone marrow cells are showed in Fig. 3b. Metaphase FISH analysis exhibited a fluorescence signal in ring chromosome 8. **c** Validation of the HOOK3-FGFR1 fusion gene with the structure of HOOK3 exons 1-11 joining to FGFR1 exons 10-18 using PCR and Sanger sequencing. **d** Graphical representation of the organization process of the formation of the HOOK3-FGFR1 fusion at the chromosome level. **e** Schematic diagrams of the HOOK3, FGFR1, and HOOK3-FGFR1 fusion proteins. The break point is indicated by the red dashed line. *CH* Calponin-homology domain, *TM* transmembrane domain

The STAR-Fusion result for HOOK3-FGFR1 fusion in Case 1 was shown in Fig. 3a (Additional file 1: Table S4). This patient showed the bilineage blasts (2.43% myeloblasts: positive for CD34; 11% monoclonal B-cell blasts: positive for CD19) at diagnosis (Fig. 2a, d). Previous studies have reported that FGFR1 fusion was concurrently observed in multiple lineages [39, 40]. We separated the BM sample into two populations (CD19^+^ and CD19^–^) by FCM on the basis of CD19 expression, and then used FISH analysis to detect the FGFR1 break-apart signal. In the CD19 negative population, we observed the split green signal consistent with a breakpoint of the FGFR1 gene in 25% cells; this was not observed in the CD19 positive population (Fig. 3b). Further, we identified 45% positive FGFR1 rearrangement signal using FISH analysis on the specimens (20.08.25) which only including myeloid blasts (Fig. 3B). These results demonstrated that FGFR1 rearrangement of Case 1 only appeared in the myeloid lineage blasts. We also used FISH analysis to confirm that the ring chromosome was chromosome 8 (Fig. 3b). Using qRT-PCR and Sanger sequencing, we further validated the HOOK3-FGFR1 fusion transcript with the breakpoints located at the 11th exon of HOOK3 and the 10th exon of FGFR1 (Fig. 3c). According to the chromosomal position, we inferred that the formation of HOOK3-FGFR1 fusion may be the result of inversion (Fig. 3d). The in-frame HOOK3-FGFR1 fusion transcript is presumed to encode a new kinase protein with 768 amino acids (Fig. 3e). The N-terminal component of the HOOK3-FGFR1 fusion protein consists of HOOK3 exons 1-11 encoding 374 amino acid residues, including a partial coiled-coil dimerization domain (Fig. 3e). The C-terminal component consists of FGFR1 exons 10-18 encoding 394 amino acid residues with only the entire tyrosine kinase domain retained, not the transmembrane (TM) domain (Fig. 3e). Based on these overall findings, we infer that the HOOK3-FGFR1 fusion protein presents constitutive activation of FGFR1 tyrosine kinase and may contribute to the pathogenesis of the Case 1.

### Activation of the NF-kappaB pathway induced by the HOOK3-FGFR1 fusion gene

**Fig. 4:**
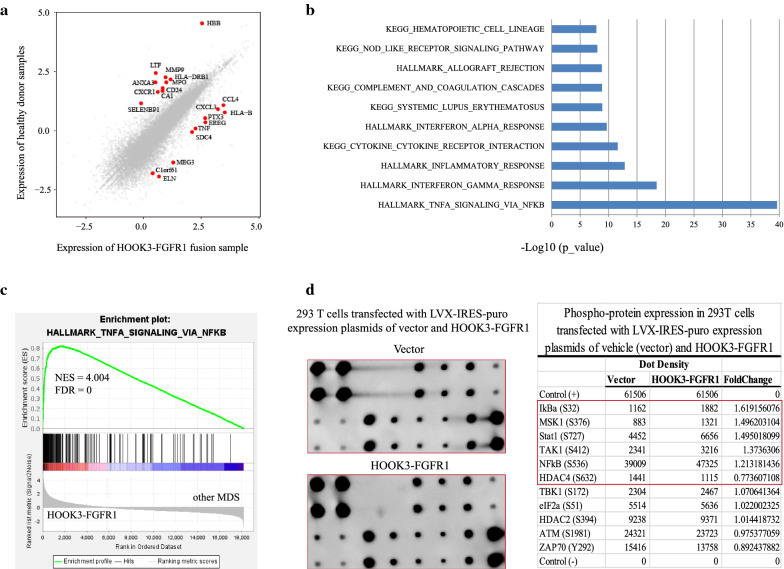
HOOK3-FGFR1 fusion gene involved in the activation of NF-kappaB signaling pathway. **a** Comparative analysis of the HOOK3-FGFR1 positive patient (Case 1) and healthy donors (n = 8). The texts in the scatterplot correspond to the top 10 up-regulated and down-regulated genes (fold change > 2 and p < 0.01). **b** Bar plot of the differentially expressed genes from the comparison of the HOOK3-FGFR1 positive patient and healthy donors (FC > 2 and Q < 0.05) enriched in MSigDB. **c** Representative GSEA plots of one HOOK3-FGFR1 positive patient compared with the 19 HOOK3-FGFR1 negative MDS patients. The normalized enrichment score (NES) and nominal p-values are shown in the graph. **d** RayBiotech NF-kappaB Pathway Phosphorylation Array including 11 proteins was used to analyze the phosphorylation status of the signaling proteins of 293 T cells transfected with LVX-IRES-puro expression plasmids of vehicle (vector) and FLAG-HOOK3-FGFR1, respectively. Visualization and quantification of the results were performed using a Typhoon 7000 phosphorimager (GE Healthcare) and NIH ImageJ software. Left panels: images of the original blots; right panel: quantitative results. The fold change in the phosphoproteins of FLAG-HOOK3-FGFR1 was calculated relative to the vector

At present, there are no data or studies describing the transcriptomic signature of FGFR1 fusion. First, we compared gene expression profiling between the HOOK3-FGFR1 fusion positive patient and 8 healthy donors. The scatterplot showed the top 10 up-regulated genes including TNF, CCL4 and CXCL3, the top10 down-regulated genes such as MMP9, ANXA3, and LTF (Fig. 4a). The functional annotation found the enrichment of HALLMARK_TNFA_SIGNALING_VIA_NFKB, and KEGG_CYTOKINE_CYTOKINE_RECEPTOR_INTERACTION pathways (Fig. 4b). Furthermore, we employed GSEA to compare the expression of the patient with HOOK3-FGFR1 fusion to the other MDS patients. We observed significantly up-regulated enrichment of HALLMARK_TNFA_SIGNALING_VIA_NFKB, PHONG_TNF_TARGETS_UP, and SANA_TNF_SIGNALING_UP (Fig. 4c and Additional file 2: Fig. S1a). These results suggest that HOOK3-FGFR1 fusion may activate the NF-kappaB signaling pathway as an unreported transcriptional feature for HOOK3-FGFR1 fusion.

Previous studies have demonstrated that the FGFR1 fusion protein plays a role in signal activation of FLT3, MYC and STAT5 [28, 41]. However, there is no study reporting activation of NF-kappaB signaling. To further validate whether HOOK3-FGFR1 fusion can trigger NF-kappaB pathway, we firstly constructed the HOOK3-FGFR1 expression vector plasmid and confirmed the presence of chimeric HOOK3-FGFR1 protein, as detected by Western blot analysis with an anti-FLAG antibody (Additional file 2: Fig. S1b). Furthermore, RayBiotech NF-kappaB pathway phosphorylation array was used to measure the phosphorylation level of NF-kappaB signaling proteins. When compared to vecter-control cells, we found that phosphorylated IKB-alpha (Ser32), TAK1 (Ser412), and NF-kappB (p65: Ser536) were increased by 1.62-fold, 1.37-fold and 1.21-fold in HOOK3-FGFR1 cells derived from 293 T cells, respectively (Fig. 4d). Taken together, these findings indicate that the HOOK3-FGFR1 fusion protein has potential to trigger NF-kappaB signaling, which may play an important role in the function of the HOOK3-FGFR1 fusion gene.

## Discussion

EMS patients characterized by FGFR1 rearrangements may be initially diagnosed as MDS [1]. In this study, we performed DNA and RNA sequencing for a cohort of 20 MDS patients and 8 healthy donors. A novel HOOK3-FGFR1 fusion was identified in one MDS patient and the predicted fusion transcript and protein were validated by PCR and Western blot, respectively. Importantly, our findings provide new evidence that the HOOK3-FGFR1 fusion gene may contribute to the pathogenesis of EMS via activation of the NF-kappaB pathway.

In general, FGFR1 rearrangement patients are characterized by the following characteristics: (i) eosinophilia; (ii) lymphoid involvement; (iii) rapid transformation; and (iv) rearrangement of 8p11 locus [1]. Our EMS patient showed coexistence of monoclonal B-lymphoid cells and myeloblasts, rearrangement of FGFR1, and aggressive progression leading to death within 3 months. However, this patient showed no evidence of eosinophilia, indicating the need to pay attention to the detection of FGFR1 fusion even in patients without eosinophilia. In addition, we confirmed that the ring chromosome is chromosome 8 (Fig. 3b). To date, ring chromosome 8 has been reported in one prostate cancer patient [42] and four AML patients [43–46]. Previous studies have reported that the presence of ring chromosomes is associated with genomic instability and leads to numerous secondary chromosome rearrangements [47]. This suggests that ring chromosome 8 may be the reason for Case 1’s complex karyotype. In this patient, HOOK3-FGFR1 fusion was formed by inversion and was barely detected by conventional karyotype analysis because the two genes were closely adjacent to each other on chromosome 8. RNA-seq has the unique ability to identify such cryptic genomic lesions and intra-chromosomal fusions. Previous studies have reported that FGFR1 translocation can present in multiple lineages [39, 40]. However, we did not detect a positive signal of FGFR1 abnormality in CD19^+^ cells in this case. Further research is needed to confirm whether HOOK3-FGFR1 can induce the involvement of multiple lineages in a mouse model.

HOOK3 has critical functions in microtubule-based motors as an adapter protein [48, 49]. Previous studies have reported that HOOK3 can serve as a fusion partner in gastrointestinal stromal tumor (GIST) and papillary thyroid carcinoma [50, 51]. Specially, FGFR1-HOOK3 fusion has been reported in GIST with the structure of FGFR1 exons 2-17 joining to HOOK3 exons 5-22 [51]. For our identified HOOK3-FGFR1 fusion gene, the breakpoints are separately located at the 11th exon of HOOK3 and the 10th exon of FGFR1 (Fig. 3c). HOOK3 protein contains one calponin-homology domain and two cytosolic coiled-coil domains. Some researchers have proposed that partner-enforced dimerization of FGFR1 is essential for EMS pathogenesis, and that the coiled-coil domain induces dimerization and activation of fusion kinases [28, 52]. The chimeric HOOK3-FGFR1 fusion protein contains the coiled-coil domain from HOOK3, indicating its potential leukemogenesis role in EMS.

FGFR1 is part of the receptor tyrosine kinase that plays crucial roles in controlling cell growth, differentiation, and survival. Specifically, FGFR1 is involved in 8p11 EMS, which is characterized by aberrant rearrangement that often produces a dimerizing protein partner fused to the kinase domain of FGFR1 [53]. It is known that FGFR1 fusion genes commonly activate downstream targets, including FLT3, MYC, and STAT5 [28, 41]. The overexpression of FGFR1 has been reported to promote NF-kappaB signaling in cancer [30, 31]. But, whether the FGFR1 fusions could induce the NF-kappaB signaling remains unclear. In this work, we observed the significantly up-regulated expression of TNF gene, further enrichment of NF-kappaB pathway in HOOK3-FGFR1 positive patient based on bioinformatics analysis (Fig. 4a-c). As we known, the tumor necrosis factor alpha (TNFα) receptor recruits the transforming growth factor β-activated kinase 1 (TAK1) [54]. Activated TAK1 then leads to the phosphorylation and degradation of IKBα, further promotes the released NF-kappaB to translocate into the nucleus and initiate target gene transcription [55]. Notably, we uncovered the elevated phosphorylation of p65, IKBα, and TAK1 in HOOK3-FGFR1 clones using the phosphorylation antibody array, demonstrating the triggering of NF-kappaB signaling (Fig. 4d).

The effectiveness of TKIs was recently investigated in cells transduced with common variants of FGFR1 fusion genes [37, 56, 57] and primary leukemic cells from patients with EMS [36, 38, 58]. To date, three different TKIs of the FGFR1 inhibitor TKI258 (dovitinib), FLT3 inhibitor PKC412 (midostaurin), and ABL1 inhibitor AP24534 (ponatinib) have demonstrated selective inhibition of the expansion of EMS cells compared to normal bone marrow cells. However, our HOOK3-FGFR1 positive patient failed to respond to ponatinib and never archived at remission (Fig. 2a). NF-kappaB was found to play a crucial role in maintenance of tumor-initiating cells (T-ICs) in leukemia [59]. Previous research has found that activation of the NF-kappaB pathway correlates with low sensitivity to bortezomib and ixazomib in the treatment of multiple myeloma [60]. NF-kappaB signaling was reported to promote the sorafenib resistance in hepatocellular carcinoma (HCC), and combined treatment with the NF-kappaB inhibitor showed increased sensitivity of HCC cells to sorafenib treatment [61]. Therefore, NF-kappaB inhibitor has potential as a combination treatment drug for FGFR1 fusion patients in the future. These findings also give us a hint that the activation of NF-kappaB signaling induced by HOOK3-FGFR1 may contribute to the failure response to ponatinib for our FGFR1 fusion patient. But, the detail mechanism remains to be explored.

## Conclusions

Patients with EMS, which is characterized by rearrangement of the FGFR1 gene, typically show poor prognosis. In this study, we identified and validated a novel HOOK3-FGFR1 fusion gene with the expression signature of activating NF-kappaB signaling. Importantly, we further provide evidence by phosphorylation antibody array that the HOOK3-FGFR1 fusion gene may contribute to the pathogenesis of EMS via activation of the NF-kappaB pathway. Given the poor outcomes for EMS cases, we hope that this finding is of potential for developing new clinical treatment for FGFR1 rearrangement patients.

## Supplementary Information

**Additional file 1:** **Table S1**. Clinical and laboratory characteristics of the 20 MDS patients. **Table S2**. A panel of 38 commonly mutated genes in myeloid hematologic malignancies. **Table S3**. Mutations detected by the target panel or WES in the MDS patients. **Table S4**. Gene fusions detected by STAR-Fusion in the 20 MDS patients. **Table S5**. Differentially expressed genes from the comparison between the HOOK3-FGFR1 positive patient and healthy donors

**Additional file 2: Figure S1**. GSEA analysis of MDS patients and western blot of 293 T cells transfected with HOOK3-FGFR1 fusion. **a** Representative GSEA plots of one HOOK3-FGFR1 positive patient compared with the 19 HOOK3-FGFR1 negative MDS patients. The normalized enrichment score (NES) and nominal p-values are shown in the graph. HOOK3-FGFR1 fusion protein was detected by western blot using anti-FLAG antibody

## Data Availability

The raw RNA-seq dataset is part of an unpublished project and is available upon request from the corresponding author (Jinsong Yan).

## Abbreviations

FGFR1: Fibroblast growth factor receptor 1
EMS: Myeloproliferative syndrome
TKI: Tyrosine kinase inhibitors
MDS: Myelodysplastic syndromes
NF-kappaB: Nuclear factor-kappaB
WHO: World Health Organization
AML: Acute myeloid leukemia
BM: Bone marrow
MNC: Mononucleated cells
NR: No remission
RC: Ring chromosome
TM: Transmembrane
DNA-seq: DNA sequencing
RNA-seq: RNA sequencing
WES: Whole exome sequencing
qRT-PCR: Quantitative real-time PCR
FCM: Flow cytometry
FISH: Fluorescence in situ hybridization
GSEA: Gene set enrichment analysis
NES: Normalized enrichment score
MSigDB: Molecular Signatures Database
RPKM: Reads Per Kilobase per Million mapped reads
NES: Normalized enrichment score
GIST: Gastrointestinal stromal tumor
HCC: Hepatocellular carcinoma
T-ICs: Tumor-initiating cells
TNFα: Tumor necrosis factor alpha
TAK1: Transforming growth factor β-activated kinase 1
CH: Calponin-homology domain
ASXH: Asx homology
ASXN: Asx N-terminal
NR box: Nuclear receptor co-regulator binding motif
PHD: Plant homeodomain
ZNF: Zinc finger
UHM: U2AF homology motif
