# Supplementary figures and images for: Identification of a novel HOOK3-FGFR1 fusion gene involved in activation of the NF-kappaB pathway
Source: Cancer Cell Int. 2022 Jan 26;22:40. doi: 10.1186/s12935-022-02451-y (PMC8793161; doi:10.1186/s12935-022-02451-y)

A

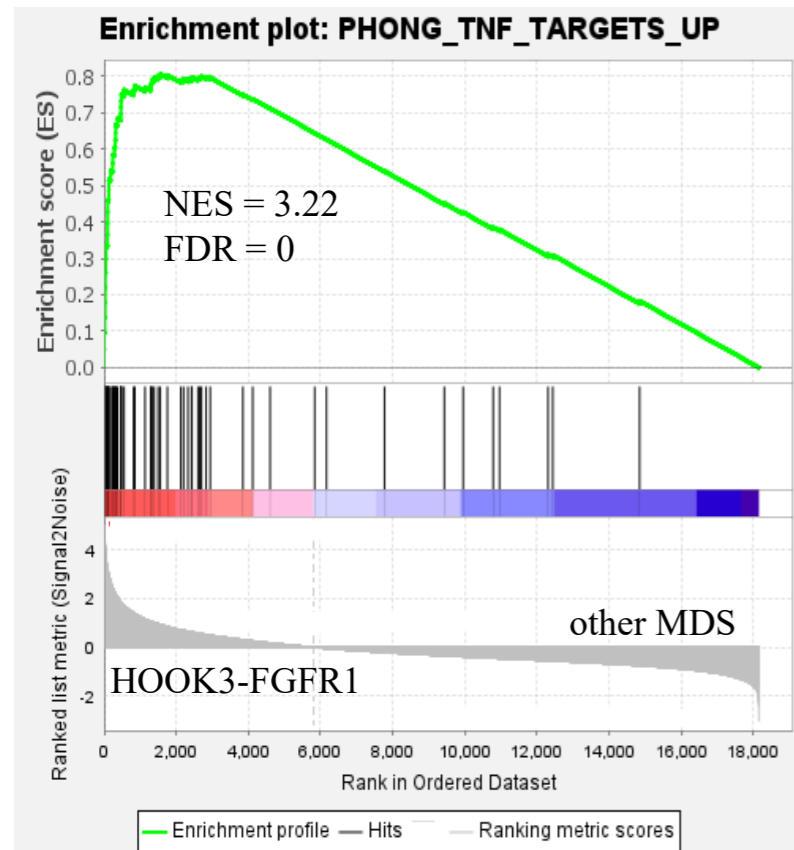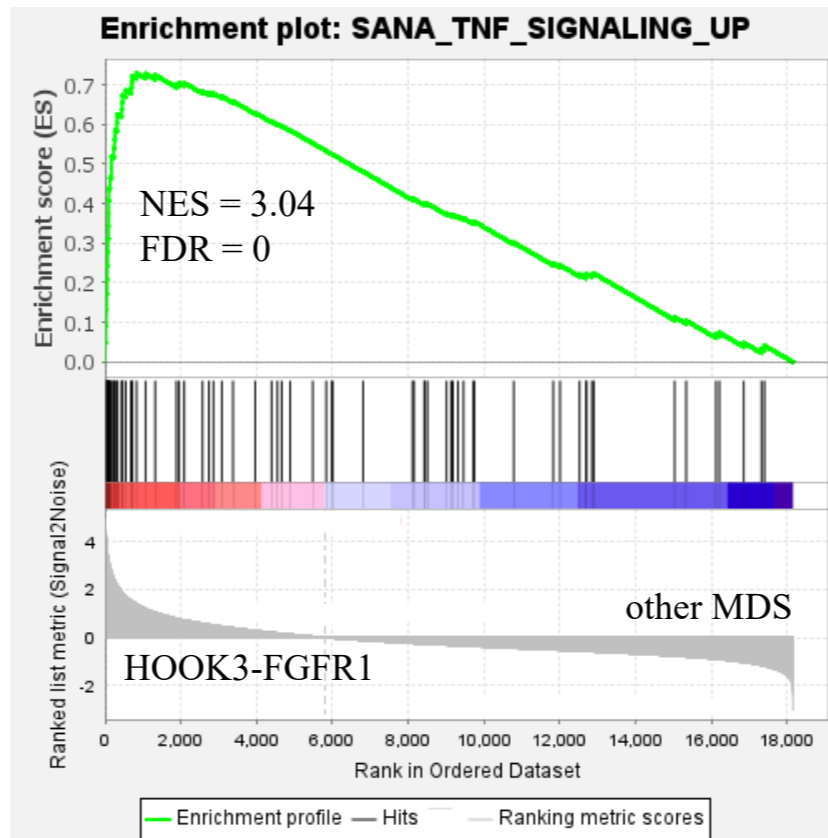

B

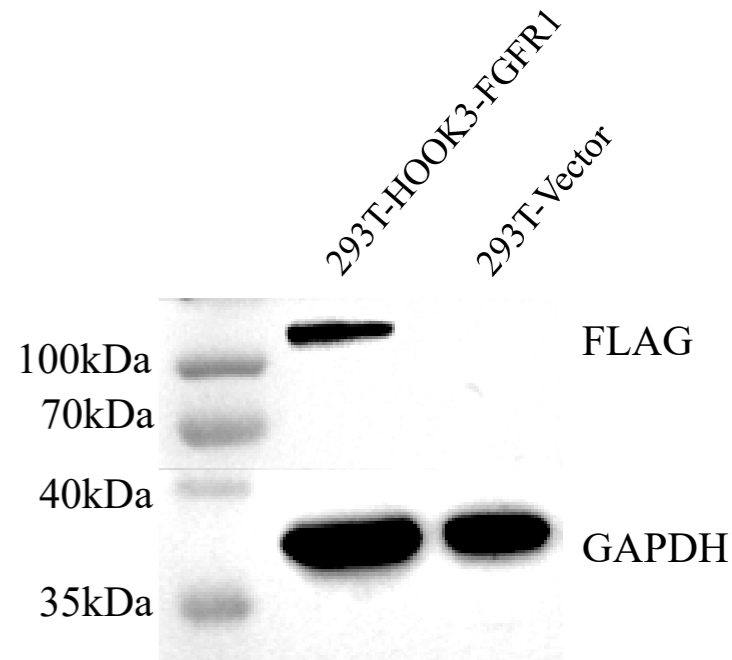

Supplement: Supplementary file 2 — Additional file 2: Figure S1. GSEA analysis of MDS patients and western blot of 293 T cells transfected with HOOK3-FGFR1 fusion. a Representative GSEA plots of one HOOK3-FGFR1 positive patient compared with the 19 HOOK3-FGFR1 negative MDS patients. The normalized enrichment score (NES) and nominal p-values are shown in the graph. HOOK3-FGFR1 fusion protein was detected by western blot using anti-FLAG antibody [file 12935_2022_2451_MOESM2_ESM.pdf]
